# Human brucellosis in the Emirate of Abu Dhabi, United Arab Emirates, 2010–2015

**DOI:** 10.1186/s12879-016-1900-9

**Published:** 2016-10-12

**Authors:** Nawal Al Shehhi, Faisal Aziz, Farida Al Hosani, Bashir Aden, Iain Blair

**Affiliations:** 1 Communicable Disease Control and Prevention Centre, Khasab Hospital, Khasab, Oman; 2 Institute of Public Health, College of Medicine and Health Sciences, United Arab Emirates University, PO Box 17666, Al Ain, United Arab Emirates

**Keywords:** Human brucellosis, *Brucella*, Incidence, United Arab Emirates, Abu Dhabi, Emirati

## Abstract

**Background:**

Worldwide, human brucellosis remains an important and widespread infection. In the past, there were limited data on the occurrence of human brucellosis in the United Arab Emirates and the reported incidence appeared to be low compared with similar areas. In 2009, a new web-based infectious disease surveillance system was introduced in the Emirate of Abu Dhabi. This paper reports data from this new system on human brucellosis for the 6 years 2010 to 2015.

**Methods:**

A dataset was extracted for each case of human brucellosis reported to the notification system for the 6 year period January 2010 to December 2015. Annual brucellosis rates by age-group, gender, nationality and, geographical region were calculated and compared.

**Results:**

A total of 480 cases of brucellosis were reported. The overall crude notification rate was 3 · 3 per 100,000 population but higher rates were seen in certain population subgroups notably expatriate males of working age in the Eastern Region (approximately 10 per 100,000) and UAE nationals of all ages and both genders in Abu Dhabi (between 4 -- 24 per 100,000).

**Conclusions:**

These findings reflect environmental and behavioral factors linked to occupation and leisure time activities associated with the large number of small non-commercial livestock farms in Abu Dhabi. Controlling human brucellosis in these circumstances will be challenging.

## Background

Globally, human brucellosis remains an important and widespread infection [1]. As a zoonosis, the occurrence of human brucellosis is largely dependent on its animal reservoir [2]. The main animal species that are affected are food-producing animals such as cattle, sheep, goats and, pigs but in some regions camels, dogs and, horses are significant sources of infection. Transmission from animal to human is usually due to the consumption of unpasteurized milk and dairy products or by direct contact, often in an occupational setting, with infected animals or their close environment, particularly at the time of parturition. There is wide between and within country variation in the occurrence of human brucellosis due to demographic and socioeconomic factors and the implementation of surveillance systems and animal-based control programs. Some countries have reduced and even eliminated brucellosis (France, Israel, Latin America) while others are seeing an emergence or re-emergence (Central Asia) [3].

In the past, there has been limited data on the occurrence of human brucellosis in the United Arab Emirates (UAE) and the reported incidence appeared to be low compared with other countries in the Region. A widely quoted 2006 study reported a rate of 4 · 1 per 100,000 population per year [3]. Although animal (sheep, goat, camels) surveillance data is sparse it is likely that in the UAE there is a significant animal reservoir of *Brucella* infection [4, 5]. A recent systematic review of human and animal brucellosis in the Middle East supported the widespread presence of *Brucella* in the region. Fifteen countries had at least one occurrence of *Brucella melitensis* and nine reported *Brucella abortus*. Four studies gave reliable estimates of brucellosis in ruminants of 2–10 % in individual animals and up to 50 % for flocks while only one study, from Egypt, reported on human brucellosis with an estimated annual incidence of 64–70/100 000 population between 2002 and 2003. Risk factors human brucellosis were consumption of unpasteurized dairy and occupational exposure. The authors concluded that although reliable data is limited, animal, and therefore, human brucellosis remains an important public health problem in all countries of the Middle East [6].

**Fig. 1:**
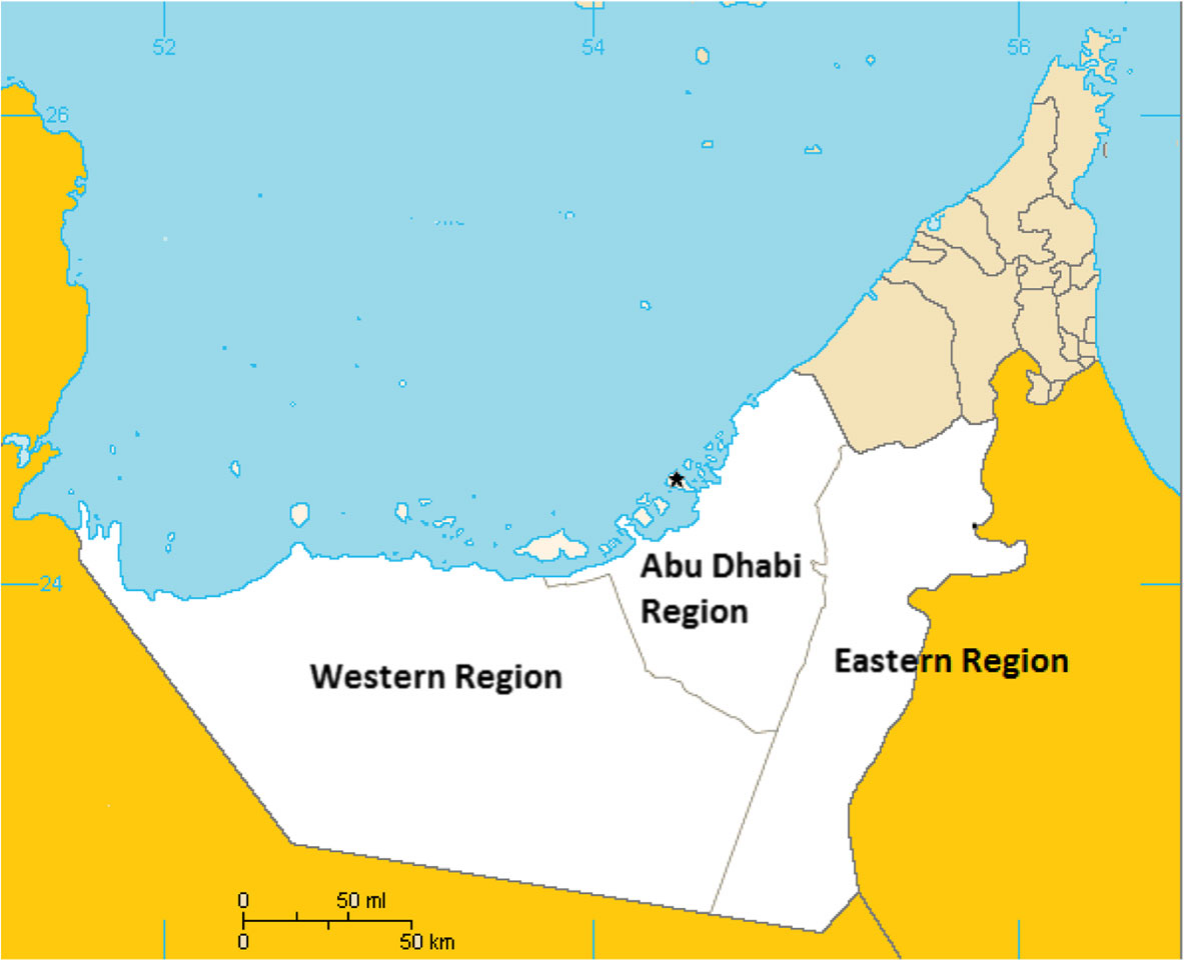
Map of the Emirate of Abu Dhabi showing the three regions. * = Abu Dhabi City

**Fig. 2:**
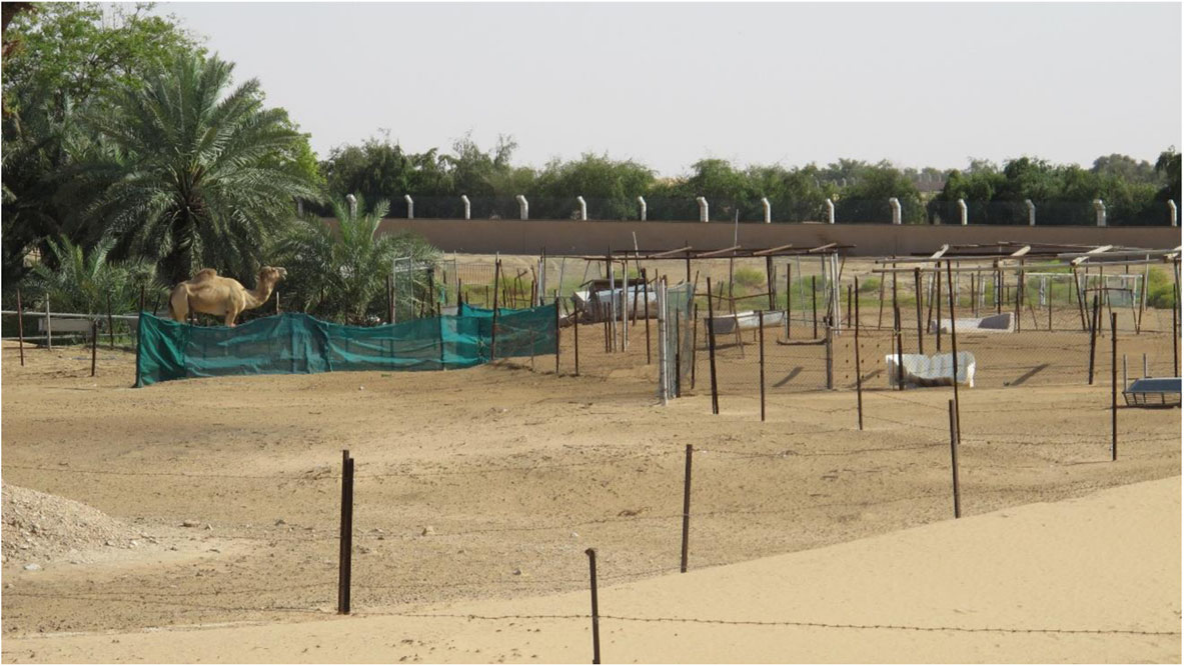
A typical livestock holding (izba) near Al Ain in the Eastern Region of Abu Dhabi

Abu Dhabi is the largest of the seven Emirates that make up the UAE having a population of 2 · 6 million and a land area of 67,000 km^2^. Abu Dhabi has three regions namely the Eastern Region, the Western Region and Abu Dhabi Region (Fig. 1). Within Abu Dhabi, many families continue the pastoral way of life of their forebears by maintaining small livestock holdings (Arabic i*zba*) in the rural areas surrounding the towns and cities (Fig. 2). These farms are often makeshift with mixed flocks of goats, sheep and, camels and provide a leisure interest and a family supply of meat and dairy products. Camels are also an important part of local tradition, camel racing is popular and is often accompanied by active camel trading [7]. Considering the popularity of raising sheep, goats and, camels and consuming their milk and dairy products, often without pasteurization, the low reported incidence of human brucellosis is therefore questionable.

A World Health Organization (WHO) commissioned review of the scientific literature on the occurrence of human brucellosis commented on the lack of reliable incidence data because passively acquired national surveillance data are likely to underestimate the true disease burden [8]. The authors recommended higher quality research and surveillance and an integration of human and animal surveillance data to ensure that the epidemiology of human brucellosis is properly defined and control programs are targeted at high burden areas and emerging foci of infection. A more recent WHO report also once again highlighted the burden of foodborne brucellosis in the Middle Eastern and African regions [9].

In 2009, a new web-based electronic system (e-notification system) replaced the former paper based infectious disease notification system in Abu Dhabi. This has improved the quality of infectious disease surveillance data in Abu Dhabi and for the first time allows the reporting of reliable data on the occurrence of human brucellosis [10]. This is the purpose of this paper in which we update estimates of the incidence of human brucellosis in Abu Dhabi for the 6 years 2010 to 2015.

## Methods

A dataset comprising date of report, age, gender, nationality, region of residence and status (Table 1) was extracted for each case of human brucellosis reported to the Health Authority Abu Dhabi (HAAD) e-notification system for the 6 year period January 2010 to December 2015.

**Table 1:** Dataset for human brucellosis notifications

Variable name	Variable value
Date of report	Date
Age	Years
Gender	Male, female
Nationality	National, expatriate
Region	Eastern, Abu Dhabi, Western
Status	Probable, confirmed

In the UAE, in official sources, health and other administrative data are typically presented according to two categories of nationality namely those who are Emirati citizens and those who are non-citizens. Citizens are also described as “nationals” and non-citizens as expatriates. This is the terminology used by HAAD and is the one that is used in this paper. The case definition that is used by the e-notification system is the same as the one that is used for surveillance by the Centers for Disease Control and Prevention [11]. As a minimum requirement, physicians will report a clinically compatible illness that is epidemiologically linked to confirmed human or animal brucellosis as a probable case. If there is definitive laboratory evidence of *Brucella* infection the case is reported as *confirmed*. All the data used in this study are anonymized so that the identity of any individual case could not be uncovered. Since the data derives from public health surveillance of a notifiable infectious disease it is judged to be exempt from institutional review board assessment.

### Statistical analysis

Descriptive statistics were used to show the number and proportions of the notified cases by year, status (probable, confirmed), age-group, gender, nationality, region and, month of report. Mid-year population estimates are available for Abu Dhabi by age group, gender, nationality and, geographical region for 2010, 2011, 2012, 2013 and, 2014 [12]. Estimates are not available for 2015 but for the purpose of this analysis have been extrapolated based on age and nationality population growth rates. Average annual brucellosis rates by age-group, gender, nationality and, geographical region have been calculated using the total number of reports as the numerator and the mid-year population as the denominator, both summed over the 6 years 2010–2015. The age-groups that are used (0–19, 20–39, 40–59 and, 60+) were chosen pragmatically to represent children and adolescents, those of young working age, middle age and, elderly. Confidence intervals for the rates were computed using a Poisson distribution. Incidence rate ratios were used to compare rates by age-group, gender, nationality and, region. For this analysis, to overcome the issue of dispersion of data, negative binomial regression was used rather than Poisson regression. Negative binomial regression models the probability that a person with a particular characteristic experiences an event (in this case infection with *Brucella*). Microsoft Excel 2010© was used for data entry and analyses were conducted using Stata (version 14). For statistical significance, 95 % CIs and *p* value < 0 · 05 were used.

## Results

**Fig. 3:**
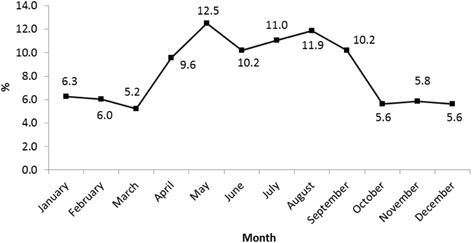
Notifications of human brucellosis (%), Abu Dhabi 2010–2015 by month of report. Source: HAAD e-notification system

In the six-year period 2010–2015, a total of 480 cases of brucellosis were reported to the HAAD e-notification system. There were more males (79 %) than females, the mean age was 30, 48 % were in the 20–39 year age group and 39 % were nationals. Fifty one percent were from Abu Dhabi Region, 42 % were from the Eastern Region and only 6 % were from the Western Region. Two hundred and ninety cases (72 %) were confirmed by laboratory testing (Table 2). Forty seven cases were reported in 2010, 75 cases were reported in 2011, 135 cases were reported in 2012, there were 99 reports in 2013, 49 reports in 2014 and, 75 reports in 2015 (Table 3). The annual number of cases peaked in 2012 but in both 2012 and 2013 a smaller proportion of cases were confirmed compared to the two earlier years. In 2014 and 2015 the proportion of confirmed cases has plateaued at about 50 %. To improve statistical precision, the six years data has been aggregated for the following analyses. Figure 3 confirms the seasonal pattern of brucellosis with reports peaking in the summer months associated with warmer weather and farming practices. The overall crude notification rate was 3 · 3 per 100,000 population. Based on 95 % confidence intervals, significantly higher rates were observed in males (3 · 8) than females (2 · 2), nationals (6 · 4) compared with expatriates (2 · 5), those aged 60 years and over (8.0) and Eastern Region residents (5 · 2). No obvious time trend was discernible (Table 4).

**Table 2:** Characteristics of notified cases of human brucellosis, Abu Dhabi 2010–2015

Characteristic	Number	Percent
Gender		
Female	101	21 · 0
Male	379	79 · 0
Age – years (Mean [SD])	30 · 5	16 · 5
Age - Categories		
0–19 years	111	23 · 1
20–39 years	232	48 · 3
40–59 years	116	24 · 2
60+ years	21	4 · 4
Nationality		
National	187	39 · 0
Expatriate	293	61 · 0
Region		
Abu Dhabi	247	51 · 5
Eastern	204	42 · 5
Western	29	6 · 0
Diagnosis		
Confirmed cases	328	68 · 3
Probable cases	152	31 · 7

Source: HAAD e-notification system

**Table 3:** Notifications of human brucellosis, Abu Dhabi 2010–2015 by year and status

	Brucellosis Cases				
	N	Confirmed		Probable	
Year		n	%	n	%
2010	47	37	78 · 7	10	21 · 3
2011	75	61	81 · 3	14	18 · 7
2012	135	96	71 · 1	39	28 · 9
2013	99	69	69 · 7	30	30 · 3
2014	49	26	53 · 1	23	46 · 9
2015	75	39	52 · 0	36	48 · 0
Total	480	328	68 · 3	152	31 · 7

Source: HAAD e-notification system

Confidence Intervals are calculated using the Poisson distribution

*Abbreviations*: *NR* notification rate, *CI* confidence interval

**Table 4:** Crude notification rates (per 100,000 population) and 95 % confidence intervals^a^, human brucellosis, 2010–2015, Abu Dhabi Emirate, United Arab Emirates

	N (%)	NR	95 % CI^a^
Overall	480	3 · 31	3 · 02–3 · 62
Gender			
Male	379 (79 · 0)	3 · 8	3 · 43–4 · 21
Female	101 (21 · 0)	2 · 23	1 · 82–2 · 71
Nationality			
Nationals	187 (39 · 0)	6 · 39	5 · 50–7 · 37
Expatriates	293 (61 · 0)	2 · 53	2 · 25–2 · 84
Age			
0–19 years	111 (23 · 1)	3 · 41	2 · 81–4 · 11
20–39 years	232 (48 · 3)	2 · 84	2 · 49–3 · 23
40–59 years	116 (24 · 2)	4 · 13	3 · 41–4 · 95
60+ years	21 (4 · 4)	8 · 03	4 · 97–12 · 27
Year			
2010	47 (9 · 8)	2 · 36	1 · 74–3 · 14
2011	75 (15 · 6)	3 · 47	2 · 73–4 · 35
2012	135 (28 · 1)	5 · 78	4 · 85–6 · 84
2013	99 (20 · 6)	4 · 04	3 · 28–4 · 91
2014	49 (10 · 2)	1 · 83	1 · 36–2 · 42
2015	75 (15 · 6)	2 · 61	2 · 05–3 · 27
Region			
Abu Dhabi	247 (51 · 5)	2 · 79	2 · 45–3 · 16
Eastern	204 (42 · 5)	5 · 22	4 · 53–5 · 99
Western	29 (6 · 0)	1 · 69	1 · 13–2 · 42

Source: HAAD e-notification system and Statistics Center Abu Dhabi

Table 5 summarises the reports by age-group, gender, nationality and geographical region of residence. Most cases were reported from the Eastern Region amongst expatriate males of working age and from Abu Dhabi Region amongst national males and females of younger age and expatriate males of working age. This pattern reflects the relative population density in these areas but additional caution is required when interpreting this geographical distribution since region of report will not necessarily be the same as the region in which infection was acquired. This may be particularly relevant for nationals who may reside in Abu Dhabi but spend leisure time in the Eastern Region (see below). Specific rates are also shown in Table 5. The highest rates are seen amongst expatriate males of working age in the Eastern Region (approximately 10 per 100,000) and amongst UAE nationals of all ages and both genders in Abu Dhabi (4–24 per 100,000). Incidence rate ratios were used to further compare the rates. The adjusted data (Table 6) suggest that being aged 40 years and over, of male gender, of Emirati nationality and resident in Abu Dhabi Region are independently associated with an increased risk of brucellosis.

**Table 5:** Human brucellosis notification rates (per 100,000 population) and 95 % confidence intervals^a^, 2010–2015, Abu Dhabi Emirate, United Arab Emirates, by gender, age-group, nationality and, region

	UAE Nationals						Expatriates					
	Male			Female			Male			Female		
	n	NR	95 % CI	n	NR	95 % CI	n	NR	95 % CI	n	NR	95 % CI
*All Regions, all ages*	122	8 · 21	6 · 82–9 · 80	65	4 · 51	3 · 48–5 · 75	257	3 · 03	2 · 67–3 · 43	36	1 · 17	0 · 09–1 · 62
*All Regions*												
0–19 years	58	7 · 87	5 · 97–10 · 17	27	3 · 89	2 · 56–5 · 65	18	1 · 89	1 · 12–2 · 99	8	0 · 92	0 · 40–1 · 81
20–39 years	33	6 · 34	4 · 37–8 · 91	16	3 · 01	1 · 72–4 · 89	167	3 · 03	2 · 59–3 · 52	16	1 · 00	0 · 57–1 · 62
40–59 years	22	12 · 72	7 · 97–19 · 26	17	9 · 94	5 · 79–15 · 92	67	3 · 52	2 · 73–4 · 47	10	1 · 79	0 · 86–3 · 28
60+ years	9	16 · 15	7 · 38–30 · 65	5	11 · 19	3 · 63–26 · 11	5	4 · 65	1 · 51–10 · 85	2	3 · 73	0 · 45–13 · 48
*Abu Dhabi Region*												
0–19 years	47	12 · 34	9 · 06–16 · 4	22	6 · 18	3 · 87–9 · 36	14	2 · 29	1 · 25–3 · 84	4	0 · 69	0 · 06–1 · 79
20–39 years	26	9 · 58	6 · 26–14 · 04	13	4 · 24	2 · 26–7 · 25	46	1 · 34	0 · 98–1 · 79	10	0 · 92	0 · 44–1 · 69
40–59 years	14	14 · 58	7 · 97–24 · 46	13	13 · 85	7 · 38–23 · 69	15	1 · 34	0 · 75–2 · 1	9	2 · 39	1 · 09–4 · 54
60+ years	7	24 · 12	9 · 70–49 · 69	4	17 · 13	4 · 67–43 · 86	2	2 · 84	0 · 34–10 · 26	1	2 · 49	0 · 63–13 · 88
*Eastern Region*												
0–19 years	9	2 · 83	1 · 30–5 · 38	5	1 · 65	0 · 53–3 · 84	4	1 · 49	0 · 41–3 · 82	1	0 · 41	0 · 01–2 · 30
20–39 years	7	3 · 47	1 · 39–7 · 15	3	1 · 50	0 · 31–4 · 39	106	9 · 14	7 · 49–11 · 06	5	1 · 14	0 · 37–2 · 66
40–59 years	8	12 · 39	5 · 35–24 · 41	4	5 · 78	1 · 57–14 · 79	47	10 · 72	7 · 88–14 · 25	1	0 · 82	0 · 02–4 · 58
60+ years	1	4 · 24	0 · 11–23 · 64	1	5 · 33	0 · 13–29 · 68	2	7 · 95	0 · 96–28 · 72	0	0	–
*Western Region*												
0–19 years	2	5 · 16	0 · 63–18 · 63	0	0	-	0	0	0 · 00–5 · 11	3	5 · 63	1 · 16–16 · 44
20–39 years	0	0	-	0	0	-	15	1 · 62	0 · 91–2 · 67	1	1 · 37	0 · 035–7 · 62
40–59 years	0	0	-	0	0	-	5	1 · 45	0 · 47–3 · 39	0	0	-
60+ years	1	31 · 77	0 · 80–176 · 99	0	0	-	1	8 · 34	0 · 21–46 · 45	1	66 · 89	1 · 69–372 · 69

Source: HAAD e-notification system and Statistics Center Abu Dhabi

*Abbreviations*: *NR* notification rate, *CI* confidence interval

^a^Confidence Intervals are calculated by using Poisson distribution

**Table 6:** Unadjusted and adjusted human brucellosis incidence rate ratios and 95 % confidence intervals ^a^, 2010–2015, Abu Dhabi Emirate, United Arab by age-group, gender, nationality and, Region

	Unadjusted negative binomial regression			Adjusted negative binomial regression		
	Brucellosis			Brucellosis		
Predictors	IRR	95 % CI	*P* Value	IRR	95 % CI	*P* Value
Age						
0–19 years	Ref			Ref		
20–39 years	0 · 95	0 · 44–2 · 05	0 · 905	1 · 17	0 · 63–2 · 17	0 · 625
40–59 years	1 · 74	0 · 79–3 · 81	0 · 168	1 · 95	1 · 04–3 · 64	0 · 037
60+ years	2 · 90	1 · 17–7 · 15	0 · 021	2 · 81	1 · 31–6 · 04	0 · 008
Gender						
Female	Ref			Ref		
Male	1 · 84	0 · 98–3 · 43	0 · 057	2 · 13	1 · 32–3 · 45	0 · 002
Nationality						
Expatriate	Ref			Ref		
Emirati National	2 · 54	1 · 45–4 · 45	0 · 001	2 · 52	1 · 55–4 · 11	<0 · 001
Region						
Western	Ref			Ref		
Abu Dhabi	3 · 04	1 · 25–7 · 38	0 · 014	2 · 09	1 · 01–4 · 35	0 · 048
Eastern	1 · 99	0 · 81–4 · 91	0 · 133	1 · 78	0 · 87–3 · 67	0 · 116

Source: HAAD e-notification system and Statistics Center Abu Dhabi

*Abbreviations*: *IRR* incidence rate ratio, *CI* confidence interval

^a^Confidence Intervals are calculated using negative binomial regression

## Discussion

In this study, we have estimated the overall crude human brucellosis notification rate for Abu Dhabi for 2010–2015 as 3 · 3 per 100,000 population per year. Our estimate updates the previously published estimate for the United Arab Emirates of 4 · 1 per 100,000 which was based on Ministry of Health data for the years 1994–2000. This new Abu Dhabi estimate is in contrast to higher rates seen in other Middle East and North African countries (Syria 160, Saudi Arabia 21, Iraq 28 and Algeria 8). Also, in the UAE in 2009, 158 brucellosis cases were reported in a population which at that time was estimated to be about 8 million. This gives a crude incidence rate of approximately 2 per 100,000 population per year which is not dissimilar to the new estimate reported here [13, 14]. However a new finding, from this study, is the higher incidence of brucellosis in certain population subgroups notably expatriate males of working age in the Eastern Region (approximately 10 per 100,000) and UAE nationals of all ages and both genders in Abu Dhabi (4–24 per 100,000). Incidence rate ratios provide further evidence for the risk factors of human brucellosis in Abu Dhabi. Aged 40 years and over, of male gender, of Emirati nationality and, resident in Abu Dhabi Region identifies a section of UAE society who are at an increased risk of brucellosis.

Our findings reflect environmental and behavioral factors linked to occupation and leisure time activities. In countries where food hygiene practices prevent foodborne brucellosis, the disease is largely occupational, and the majority of cases are seen in males between the ages of 20 and 45 years. However, in countries where untreated dairy products are consumed the wider population is at risk and cases can occur in women and children. Both of these factors explain the distribution of cases reported here. Livestock keeping, particularly of small ruminants such as sheep and goats in which *Brucella* infection is prevalent, is popular amongst Emirati families who seek to preserve their pastoral heritage. Indeed in Abu Dhabi, in 2014, there were 24,000 livestock holdings accommodating 3 · 4 million sheep, goats and, camels [15]. Of these over half (15,000) are in the Eastern Region which traditionally has been the UAE center of agricultural activity. Many of these farms are small and makeshift with mixed herds and low standards of hygiene. There is little official data on animal brucellosis in UAE [16] but a serological survey carried out in 2009, in the three regions of Abu Dhabi, found that the prevalence of *Brucella* antibodies varied from 5.3 to 10.7 % in sheep and goats and 4.8–8.8 % in camels [17]. Opportunities for human transmission occur when herdsmen (invariably expatriate men of working age) handle and feed animals and assist with parturition and when owners (Emiratis) and their families have contact with animals during visits or consume unpasteurized milk and other food products. Our data also shows a seasonal variation in the incidence of human brucellosis with most cases occurring in the spring and summer. This period coincides with animal breeding cycles and contrasts with tropical and subtropical areas, where animal breeding can extend throughout the year and a seasonal pattern of human disease is not seen.

Our findings must be interpreted in light of the acknowledged limitations of this study. Cases of brucellosis are reported passively by clinicians and therefore, as with all such surveillance systems, are subject to under-reporting. Also not all cases are confirmed by appropriate laboratory tests so that misclassification can occur. That said, the web-based e-notification system maximizes engagement of clinicians and gives the best possible prospect of thorough ascertainment of the numerators while accurate population estimates are available for use as the denominators. The e-notification system attempts to collect a detailed dataset for each case of brucellosis but this is not always completed by the notifying clinician and this missing data means that we have not been able to report other correlates of infection such as occupation, specific risk behaviors, travel, clinical features and outcomes. Certainly, improving the completeness of our dataset would add to the understanding of the risk factors for human brucellosis in Abu Dhabi. However passive surveillance systems are prone to interviewer bias and alternative research design may be more appropriate [18].

Control of human brucellosis depends on the control of animal brucellosis by a combination of vaccination and testing and slaughter of infected animals along with better farm biosecurity and hygiene [19]. Cattle brucellosis has been eliminated in some countries but the control of brucellosis in small ruminants such as sheep and goats is more challenging. In Abu Dhabi it is not clear how this can be achieved given the very large number of small non-commercial farms and the enduring popularity amongst Emiratis of continuing the traditional pastoral way of life, spending time on their farms, looking after their animals and consuming untreated milk and dairy products. Indeed, over the six years covered by this study there is no sign of a downwards trend in human brucellosis rates.

## Conclusion

In some sub-groups of the Abu Dhabi population, brucellosis is a significant infection with rates in excess of 10 per 100,000 population per year. These are lower than the historical rates reported from some neighboring countries but are higher than might be expected based on past local surveillance data. These updated estimates have been made possible by better quality data from a new e-notification system. The data shows that some sub-sections of the population are at increased risk of brucellosis because of increased exposure due to occupation or leisure related activities. The popularity of small non-commercial livestock farms with Emirati families is unique and will challenge any efforts to introduce control measures to eliminate *Brucella* infection from livestock in Abu Dhabi.

## Abbreviations

HAAD: Health Authority Abu Dhabi
UAE: United Arab Emirates
WHO: World Health Organization
